# CX_3_CL1 is up-regulated in the rat hippocampus during memory-associated synaptic plasticity

**DOI:** 10.3389/fncel.2014.00233

**Published:** 2014-08-12

**Authors:** Graham K. Sheridan, Anita Wdowicz, Mark Pickering, Orla Watters, Paul Halley, Niamh C. O’Sullivan, Claire Mooney, David J. O’Connell, John J. O’Connor, Keith J. Murphy

**Affiliations:** ^1^Neurotherapeutics Research Group, UCD School of Biomolecular and Biomedical Science, Conway Institute, University College DublinDublin, Ireland; ^2^Department of Physiology, Development and Neuroscience, University of CambridgeCambridge, UK; ^3^School of Medicine and Medical Science, Health Sciences Centre, University College DublinDublin, Ireland; ^4^UCD School of Biomolecular and Biomedical Science, Conway Institute, University College DublinDublin, Ireland

**Keywords:** calcium imaging, chemokine signaling, fractalkine, learning and memory, LTP, water maze

## Abstract

Several cytokines and chemokines are now known to play normal physiological roles in the brain where they act as key regulators of communication between neurons, glia, and microglia. In particular, cytokines and chemokines can affect cardinal cellular and molecular processes of hippocampal-dependent long-term memory consolidation including synaptic plasticity, synaptic scaling and neurogenesis. The chemokine, CX_3_CL1 (fractalkine), has been shown to modulate synaptic transmission and long-term potentiation (LTP) in the CA1 pyramidal cell layer of the hippocampus. Here, we confirm widespread expression of CX_3_CL1 on mature neurons in the adult rat hippocampus. We report an up-regulation in CX_3_CL1 protein expression in the CA1, CA3 and dentate gyrus (DG) of the rat hippocampus 2 h after spatial learning in the water maze task. Moreover, the same temporal increase in CX_3_CL1 was evident following LTP-inducing theta-burst stimulation in the DG. At physiologically relevant concentrations, CX_3_CL1 inhibited LTP maintenance in the DG. This attenuation in dentate LTP was lost in the presence of GABA_A_ receptor/chloride channel antagonism. CX_3_CL1 also had opposing actions on glutamate-mediated rise in intracellular calcium in hippocampal organotypic slice cultures in the presence and absence of GABA_A_ receptor/chloride channel blockade. Using primary dissociated hippocampal cultures, we established that CX_3_CL1 reduces glutamate-mediated intracellular calcium rises in both neurons and glia in a dose dependent manner. In conclusion, CX_3_CL1 is up-regulated in the hippocampus during a brief temporal window following spatial learning the purpose of which may be to regulate glutamate-mediated neurotransmission tone. Our data supports a possible role for this chemokine in the protective plasticity process of synaptic scaling.

## INTRODUCTION

While higher inflammatory and pathophysiological levels of cytokines are implicated in a range of neuropsychiatric and neurodegeneration diseases, it is now equally evident that, within the central nervous system (CNS), cytokines, including the chemoattractant cytokines (chemokines), mediate physiological signaling functions far beyond and independent of their traditional roles in inflammation and disease (Hopkins and Rothwell, 1995; Rothwell and Hopkins, 1995; Reichenberg et al., 2001; Pollmächer et al., 2002; Wilson et al., 2002; Adler and Rogers, 2005; Adler et al., 2006; McAfoose and Baune, 2009; Hoshiko et al., 2012; Williamson and Bilbo, 2013). In particular, pro-inflammatory cytokines such as interleukin (IL)-1, IL-6, and tumor necrosis factor alpha (TNF-α) have all been implicated in cardinal cellular and molecular processes of long-term hippocampal-dependent memory consolidation including synaptic plasticity, synaptic scaling and neurogenesis (Malenka and Bear, 2004; Bruel-Jungerman et al., 2007a,b; Turrigiano, 2007; Baier et al., 2009; McAfoose and Baune, 2009; Bachstetter et al., 2011; Ben Menachem-Zidon et al., 2011; Yirmiya and Goshen, 2011; del Rey et al., 2013; Gemma and Bachstetter, 2013). For example, at physiological levels, IL-1 promotes long-term potentiation (LTP), a widely employed electrophysiological model of memory-associated synaptic plasticity, whereas IL-6 appears to exert inhibitory influences on excessive excitation during LTP maintenance (Li et al., 1997; Coogan et al., 1999; Ross et al., 2003; Balschun et al., 2004). Interestingly, production of both IL-1 and IL-6 is increased following LTP induction, further supporting a role for cytokines in modulating memory-associated synaptic plasticity and network-protective synaptic scaling (Schneider et al., 1998; Jankowsky et al., 2000). Our aim in the current study was to assess the effects of the chemokine, CX_3_CL1, also known as neurotactin or fractalkine, on hippocampal-dependent synaptic plasticity processes such as spatial memory and LTP. CX_3_CL1 is highly expressed on hippocampal neurons in the post-natal and adult rat and declines in old age which has been linked to cognitive decline in rodents (Lyons et al., 2009). In this study, we investigated if CX_3_CL1 plays a normal physiological role in hippocampal-dependent synaptic plasticity.

Several chemokines are widely expressed throughout the CNS during development and throughout life where they have been shown to play diverse roles in cell migration and differentiation (Lu et al., 2002), regulation of cellular communication in the adult brain (Tran and Miller, 2003) and neuroprotection (Araujo and Cotman, 1993; Meucci et al., 1998; Robinson et al., 1998; Bruno et al., 2000; Limatola et al., 2000; Hatori et al., 2002; Deiva et al., 2004; Krathwohl and Kaiser, 2004; Limatola et al., 2005; Catalano et al., 2013; Shepherd et al., 2013). CX_3_CL1 is the only member of the chemokine δ subfamily (Rostene et al., 2007). Most other chemokines bind several G protein-coupled receptors to mediate their activities and so CX_3_CL1 is unusual in that it appears to bind only one receptor, the G_i_ protein-coupled receptor, CX_3_CR1 (Allen et al., 2007). The full-length molecule is also larger than most other chemokines, containing approximately 373 amino acid residues compared to the more common 70–80 amino acid size. The 95 kDa full-length protein is a type I transmembrane protein consisting of a 76-amino acid *N*-terminal chemokine domain, a 241-amino acid glycosylated mucin-like stalk, an 18-amino acid transmembrane region and an intracellular *C*-terminal domain. The approximately 70 kDa soluble *N*-terminal chemokine domain can be released from the full-length protein via the action of several metalloproteinases such as cathepsin S, ADAM10, and ADAM17 (TACE: TNF-α-converting enzyme) in both the periphery and CNS (Garton et al., 2001; Hundhausen et al., 2003; Clark et al., 2007; Cook et al., 2010; Jones et al., 2013). Unlike most chemokines, CX_3_CL1 is constitutively expressed in the CNS with particularly high levels in hippocampal neurons (Harrison et al., 1998). CX_3_CR1, the only known receptor for CX_3_CL1, is expressed predominantly on microglia in the mouse CNS (Cardona et al., 2006). The cell type expression pattern of CX_3_CR1 in the CNS remains controversial, however, since several studies report CX_3_CR1 expression on neurons *in vitro* as well as in brain regions including the hippocampus, Raphe nucleus, nucleus of the solitary tract (NTS) and paraventricular nucleus (PVN) of the hypothalamus in rats (Meucci et al., 1998, 2000; Maciejewski-Lenoir et al., 1999; Hatori et al., 2002; Hughes et al., 2002; Tarozzo et al., 2003; Verge et al., 2004; Limatola et al., 2005; Zhuang et al., 2007; Heinisch and Kirby, 2009; Ruchaya et al., 2012, 2014).

The high basal level of CX_3_CL1 mRNA and protein expression in the hippocampus is suggestive of a physiological, non-inflammatory function. Indeed, there is mounting evidence which implicates both CX_3_CL1 and its receptor, CX_3_CR1, in synaptic plasticity and neuromodulation (Bertollini et al., 2006; Ragozzino et al., 2006; Piccinin et al., 2010; Maggi et al., 2011; Rogers et al., 2011; Roseti et al., 2013; Scianni et al., 2013). For example, CX_3_CL1 has been shown to reduce spontaneous glutamate release and post-synaptic glutamate currents (Meucci et al., 1998; Limatola et al., 2005). The latter effect has been linked to increased intracellular calcium and dephosphorylation of the GluR1 AMPA receptor subunit (Ragozzino et al., 2006). These synaptic effects are consistent with a direct action of CX_3_CL1 on neurons most likely exerted through the CX_3_CR1 receptor, which is reportedly expressed on the dendrites of hippocampal neurons (Meucci et al., 2000; Limatola et al., 2005). Overall, previous studies indicate a predominantly inhibitory role for CX_3_CL1, perhaps as a component of neuroprotective synaptic scaling mechanisms necessary for hippocampal memory-associated synaptic plasticity processes (Bertollini et al., 2006; Turrigiano, 2008; Piccinin et al., 2010). Consistent with this hypothesis, ADAM17-mediated increase in soluble CX_3_CL1 is observed in multiple settings of glutamatergic neurotransmission where the chemokine is suggested to perform a neuroprotective function (Chapman et al., 2000; Tsou et al., 2001; Erichsen et al., 2003; Limatola et al., 2005; Ragozzino et al., 2006; Lauro et al., 2010; Pabon et al., 2011). At levels reached during inflammatory conditions, CX_3_CL1 signaling has previously been associated with activation of pro-survival and anti-apoptotic pathways through phosphorylation of molecules such as Akt, as well as activation of MAP kinases such as p-38 and Erk1/2 (p44/42; Maciejewski-Lenoir et al., 1999; Meucci et al., 2000; Cambien et al., 2001; Deiva et al., 2004; Klosowska et al., 2009; Lyons et al., 2009).

In the present study, we investigated if CX_3_CL1 expression is actively regulated in the hippocampus during a normal spatial learning event and also after the induction of LTP. We demonstrate the ability of physiological levels of CX_3_CL1 to inhibit the maintenance of LTP and the importance of dentate gyrus (DG) GABAergic neurotransmission to facilitating this attenuation of hippocampal synaptic plasticity. Finally, we provide evidence that the effects of CX_3_CL1 on synaptic plasticity may relate to suppression of glutamate-mediated calcium influx, particularly in hippocampal neurons.

## MATERIALS AND METHODS

### ANIMAL MAINTENANCE AND BEHAVIORAL ASSESSMENT

Postnatal day 80 male Wistar rats (330–380 g) were used for behavioral studies and were obtained from the Biomedical Facility at University College Dublin, Ireland. All experimental procedures were approved by the Animal Research Ethics Committee of the Biomedical Facility at UCD and were carried out by individuals who held the appropriate license issued by the Minister for Health and Children. Animals were housed in groups of four and given *ad libitum* access to food and water. The experimental room was kept on a 12 h light/dark cycle at 22 ± 2°C. The behavior of each animal was assessed in an open field apparatus (620 mm long, 620 mm wide, and 150 mm high) both 48 and 24 h prior to commencement of training. The base of the open field box was demarcated into an 8 × 8 grid. The animals’ locomotion, rearing, and grooming behavior was monitored over a 5 min period and deemed normal prior to water maze training (data not shown). Their weights were also recorded immediately following the open field. Behavioral assessment was conducted in a quiet room under low-level red light illumination.

### WATER MAZE TRAINING

On postnatal day 80, animals were trained in the Morris water maze spatial learning task. Briefly, the water maze apparatus consists of a large circular pool (150 cm diameter, 80 cm deep) and a hidden platform (11 cm diameter). Both were constructed from black polyvinyl plastic, offering no intramaze visual cues that may help guide escape behavior. The platform was submerged 1.5 cm below the water surface (temperature 26 ± 1°C) and positioned 30 cm from the edge of the maze wall. The platform remained in the same position throughout the training session. The experimental room contained several extra-maze visual cues. The rat was lowered into the water facing the wall of the maze (30 cm high) at one of three locations which were alternated with each trial. Trials lasted a maximum of 90 s and the length of time taken for the rat to find the hidden platform was recorded. Rats failing to find the platform within the 90 s were placed on it for 10 s and allowed orient themselves. The training session consisted of five trials with an inter-trial interval of 300 s. Each trained animal was assigned a corresponding passive control animal that spent the same lengths of time swimming in the pool, minus the platform. After training, the rats were dried-off and placed back into their home cages. They were then killed by cervical dislocation at specific time-points post-training, i.e. 1, 2, or 3 h after commencement of the third trial. Brains used for immunofluorescent labeling procedures were quickly dissected out, covered in optimal cutting temperature (OCT compound; Agar Scientific) and snap frozen in *N*-hexane cooled to –80°C with CO_2_.

### IMMUNOFLUORESCENT LABELING OF HIPPOCAMPAL CX_3_CL1

Coronal cryosections of whole brain were taken at –3.3 mm with respect to Bregma in order to examine the dorsal hippocampus (Paxinos and Watson, 2005). The 12 μm sections were adhered to glass slides coated with poly-L-lysine. Sections were fixed in 70% ethanol for 25 min and then washed in phosphate-buffered saline (PBS). Sections were then incubated for 18 h at room temperature in primary antibody solution. The primary antibodies used were: (1) AF537 (R&D Systems; 1:250 dilution), a goat IgG polyclonal antibody that labels recombinant rat CX_3_CL1 and; (2) MAB377 (Millipore; 1:500 dilution), a mouse IgG monoclonal antibody that detects the neuronal marker NeuN. The primary antibody solution consisted of 1% bovine serum albumin (BSA) and 1% normal rabbit serum in PBS. Following two 10 min washes in PBS, sections were incubated for 3 h with a rabbit anti-goat IgG secondary antibody conjugated to FITC (Sigma; 1:1000 dilution) which detected the CX_3_CL1 antibody. The NeuN primary antibody was detected by a rabbit anti-mouse IgG TRITC-labeled secondary antibody (Sigma; 1:1000 dilution). Where applicable, nuclei were visualized using either propidium iodide or Hoechst 33258 (Invitrogen). Sections were mounted in Citifluor glycerol PBS solution (Agar Scientific), cover-slipped and stored in darkness at 4°C until imaged. To minimize any potential confounder effects from the immunohistochemical technique, trained sections were prepared, stained and imaged at the same time as their relevant passive control.

### ACUTE HIPPOCAMPAL SLICE PREPARATION AND fEPSP RECORDING

Post-natal day 21–25 male Wistar rats (60–100 g) were obtained from the Biomedical Facility, University College Dublin, Ireland. All experimental procedures were approved by the Animal Research Ethics Committee of the Biomedical Facility at UCD. Animals were anesthetized using isoflurane (Abbott Laboratories Ireland Ltd.) and decapitated by guillotine. The brain was rapidly removed and placed into ice-cold artificial cerebro-spinal fluid (aCSF) bubbled with 95% O_2_ and 5% CO_2_ (aCSF composition: 120 mM NaCl, 26 mM NaHCO_3_, 1.25 mM NaH_2_PO_4_, 2.5 mM KCl, 2 mM MgSO_4_⋅7H_2_0, 2 mM CaCl_2_ and 10 mM D-glucose). This high magnesium aCSF facilitates slice viability and recovery through greater NMDA receptor blockade. Transverse hippocampal slices (400 μm) were cut from both hemispheres using a vibroslice (Campden Instruments). They were then transferred to a submerged incubation chamber containing bubbled, room temperature aCSF and allowed to recover for 90 min. Following this recovery period, slices were transferred to a recording chamber perfused with aCSF at a flow rate of 4–5 ml/min at 31 ± 0.5°C. The composition of aCSF used for recording was a modified version of that used during slice recovery, i.e., the MgSO_4_⋅7H_2_O content was reduced to 1.3 mM to decrease NMDA receptor blockade and facilitate LTP induction. Extracellular field excitatory post-synaptic potentials (fEPSPs) were elicited by stimulation of the medial perforant path of the DG by a monopolar glass electrode at a frequency of 0.05 Hz. Responses were recorded using a glass electrode placed in the middle third of the molecular layer and stimulus strength was adjusted to give a response 35% of maximal. The effect of 500 pM rCX_3_CL1 (recombinant rat CX_3_CL1, Peprotech EC) on LTP in the DG was investigated both in the presence and absence of 100 μM of the GABA_A_ receptor/chloride channel inhibitor, picrotoxin (Sigma–Aldrich). Stable baseline recordings were made for at least 20 min prior to application of drugs. LTP was induced by theta-burst stimulation (TBS) consisting of eight trains (40 ms duration) of eight pulses at 200 Hz with 2 s intervals between trains and at a stimulus strength corresponding to 70% of maximal. Following TBS, the stimulus voltage was returned to that of baseline levels and fEPSPs were recorded every 20 s for a further 60 min.

### FREEZING OF HIPPOCAMPAL SLICES FOR IMMUNOSTAINING

Slices were coated in OCT (Agar UK) and snap-frozen in *n*-hexane cooled to –80°C with compressed CO_2_. Hippocampal slices were cryosectioned into 12 μm sections that were adhered to glass slides and immunofluorescently stained for CX_3_CL1 (as above). Nuclei were counterstained with propidium iodide.

### PREPARATION OF ORGANOTYPIC HIPPOCAMPAL SLICE CULTURES

Organotypic hippocampal cultures were prepared according to Stoppini et al. (1991). Briefly, post-natal day 7 male Wistar rats were decapitated without anaesthetic and their brains quickly dissected out and placed into ice-cold Earle’s balanced salt solution (EBSS) for 1 min. Both hippocampi were removed and cut into 400 μm slices using a McIlwain tissue chopper. Slices were separated and arranged onto organotypic inserts (three per insert, Millicell PICMORG50). The inserts were housed in standard six-well cell culture plates which were kept in an incubator at 35°C and 5% CO_2_ in air. The slices were grown using an interface method with 1 ml organotypic medium supplying the under-surface of the slice. The organotypic medium consisted of 50% minimum essential medium (MEM, Gibco), 25% EBSS (Gibco), 25% heat-inactivated horse serum (Sigma) and supplemented with 2 mM glutamine, 28 mM D-glucose, 100 U/ml penicillin/streptomycin, and 25 mM HEPES. The first medium change was conducted 24 h following slice preparation with subsequent medium changes occurring every 2 days. Slices were maintained for 21 days *in vitro* (DIV) prior to experimentation.

### CONFOCAL MICROSCOPY

All confocal images used for quantitative analysis of immunofluorescence (12-bit; 1024 × 1024 pixels) were captured using a 40×/0.8 W water-dipping lens (Zeiss Achroplan). Images of hippocampal sections from water maze-trained animals were captured from three defined regions of the hippocampus, i.e., CA1, CA3, and the apex of the DG. Images taken from acute slice preparations were captured from the upper (unstimulated) and lower (TBS-stimulated) blades of the DG. The specific areas of the hippocampal neuronal circuit captured were kept consistent between sections. Three sections from each rat brain and acute hippocampal slice were used for analysis.

### IMAGE ANALYSIS

Image analysis was conducted using EBImage; a package for the R programming environment (Pau et al., 2010). Analysis of the combined nuclear and surrounding cell soma expression of CX_3_CL1 fluorescence was calculated for every cell in each image. Briefly, taking the DG confocal image in Figure S1 as a typical example, the red, green and blue channels were first separated for each image and every pixel within the images (1024 × 1024) was assigned an intensity value between 0 and 1. Using the blue channel as a nuclear reference, size and fluorescence intensity thresholds were set in order to select only those pixels likely to represent Hoechst-labeled nuclei. The nuclei were then ‘dilated’ using morphological kernel expansion. This step allowed the designation of a soma region surrounding each nucleus. A distance map was then generated for the image which calculates the distance of each foreground pixel (white) to the nearest background pixel (black). The watershed segmentation algorithm is then employed which accurately separates clusters of nuclei that are very close together, or touching, into individual cells. Minimum distance between objects and minimum radius criteria are written into the analysis scripts which further refines object separation. CX_3_CL1 immunofluorescence is calculated for every cell as the average pixel fluorescence intensity.

### LIVE-CELL CALCIUM IMAGING IN ORGANOTYPIC SLICES AND PRIMARY HIPPOCAMPAL CULTURES

At 21 days *in vitro*, organotypic hippocampal cultures were prepared for calcium imaging experiments by transferring inserts to room-temperature BSS (buffered salt solution) composed of 130 mM NaCl, 5.4 mM KCl, 1.8 mM CaCl_2_, 2 mM MgSO_4_, 5.5 mM D-glucose, and 20 mM HEPES, pH 7.3. The insert membranes were cut using a scalpel and individual slices were transferred to 35 mm Petri dishes containing 2 ml of 3 μM fluo-4 AM calcium indicator (Invitrogen) in BSS and allowed incubate in the dark for 30 min. Similarly, cover-slips containing mixed neuronal-glial cultures were individually transferred to standard six-well plates, containing 3 μM fluo-4 AM in BSS, for 20 min.

Time-series calcium imaging experiments in organotypic slices were conducted in the CA1 pyramidal cell layer of the hippocampus using an upright LSM V Pascal confocal microscope (Zeiss). The field of view on the 10× water immersion lens was halved allowing us to monitor calcium responses at a rate of two frames per second. We measured baseline calcium levels in the first 20 s (i.e. average of 40 frames). After 20 s a solution of glutamate (30 μM) in BSS was washed onto the slice and filled the slice chamber. This glutamate solution remained in the chamber for 105 s. The calcium response of each cell over the 125 s time-course was calculated using EBImage software. The concentrations of rCX_3_CL1 (500 pM for 15 min) and picrotoxin (100 μM for 15 min) used for live-cell calcium imaging experiments in organotypic slices was the same as those used in acute slice electrophysiology experiments.

For live-cell calcium imaging experiments in primary mixed neuron-glial cultures, cells were loaded with 3 μM fluo-4 AM calcium indicator (Invitrogen) in BSS as above, and were then transferred to a custom-built imaging chamber containing fresh BSS. The imaging chamber allowed for wash-in/out of CX_3_CL1 and glutamate solutions (Pickering et al., 2008). Experiments were conducted at room temperature using an upright LSM V Pascal confocal microscope and a 10×/0.3 W Ph1 water-dipping lens so as to capture several hundred cells per experiment. Time-series confocal images were captured at frame-rate of 1 Hz. Cells were pre-treated for 15 min with either 500 pM or 2 nM rCX_3_CL1. Baseline calcium levels for every cell were monitored for 20 s prior to a 30 μM glutamate exposure. This glutamate solution remained in the chamber for 90 s before being washed out with fresh BSS solution and frames were captured for a further 40 s. The calcium response of each cell was calculated every second for 150 s using EBImage software.

### STATISTICAL ANALYSIS

All raw data was imported into GraphPad Prism 6 software where statistical analyses were performed. Kruskal–Wallis non-parametric analysis of variance (ANOVA) tests were performed in conjunction with Dunn’s multiple comparisons *post hoc* analyses in order to determine statistical significance (*p* < 0.01) for: (1) CX_3_CL1 regulation post-spatial learning in rats; (2) CX_3_CL1 regulation post-theta burst stimulation in acute hippocampal slices and; (3) glutamate-mediated calcium responses in primary mixed neuron-glial cell cultures. One-way ANOVA tests were performed in conjunction with Bonferroni *post hoc* analyses in order to determine statistical significance (*p* < 0.05) for: (1) the reduction in swim-times during water maze training; and (2) the effect of rCX_3_CL1 on LTP and paired pulse ratio. Mann–Whitney *U* tests were performed in order to determine statistical significance (*p* < 0.001) for the effects of rCX_3_CL1 and picrotoxin on glutamate-mediated calcium responses in organotypic hippocampal slice cultures. The relationship between NeuN expression and CX_3_CL1 expression in the rat hippocampus was analyzed by Pearson correlation and linear regression.

## RESULTS

### CX_3_CL1 IS HIGHLY EXPRESSED BY NEURONAL CELL TYPES IN THE ADULT RAT HIPPOCAMPUS

**FIGURE 1:**
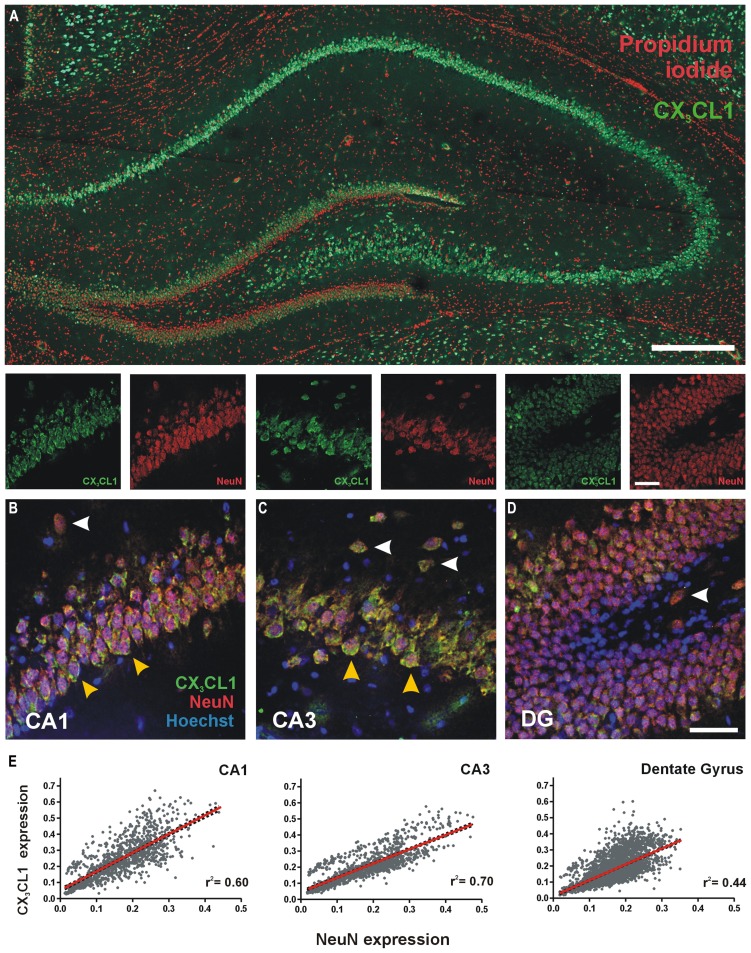
**Expression of CX_3_CL1 in the CA1, CA3 and dentate gyrus (DG) of the rat hippocampus. (A)** Basal levels of CX_3_CL1 expression in the rat dorsal hippocampus. Green: CX_3_CL1; Red: propidium iodide-stained nuclei. Scale bar = 200 μm. **(B–D)** CA1, CA3 and DG regions of the hippocampus, respectively, showing CX_3_CL1 (green), NeuN (red) and Hoechst (blue) expression. White arrowheads: interneurons; yellow arrowheads: CA pyramidal neurons. **(E)** Relationship between CX_3_CL1 and NeuN expression. Each dot represents a single cell. Linear regression analyses (red lines) appear on each plot with corresponding *r*^2^ values indicated. Linear regression *p* < 0.05 for CA1, CA3 and DG regions of the hippocampus.

We characterized the expression and distribution of CX_3_CL1 in the rat dorsal hippocampus using immunofluorescence. CX_3_CL1 expression was predominately restricted to the glutamatergic pyramidal and granule neurons of the hippocampus (**Figure 1A**). At higher magnification, it is clear that CX_3_CL1 expression co-localizes with the neuronal marker NeuN in CA1 and CA3 pyramidal neurons (**Figures 1B,C**; yellow arrowheads), dentate granule cells (**Figure 1D**) and presumptive interneurons outside the primary cell layers in all three hippocampal subfields (**Figures 1B–D**; white arrowheads). In all cases, the chemokine appears to be predominantly expressed on the plasma membrane of the cell soma. We assigned expression intensity values for NeuN and CX_3_CL1 to each cell in the image dataset (see Figure S1 for image analysis method) and then analyzed the relationship between expression of the mature neuronal marker, NeuN, and CX_3_CL1 by Pearson correlation in each sub-region of the hippocampus. A high degree of correlation was evident between CX_3_CL1 and NeuN expression in all three regions of the hippocampus (**Figure 1E**). The CA3 region showed the strongest linear regression coefficients of determination for correlation between CX_3_CL1 and NeuN expression (*r*^2^ = 0.70, *p* < 0.05). These data confirm previous reports that mature NeuN + hippocampal neurons in the adult rat hippocampus express high levels of CX_3_CL1 under naïve resting conditions.

### CX_3_CL1 IS UP-REGULATED 2 h POST-SPATIAL LEARNING IN THE RAT HIPPOCAMPUS

**FIGURE 2:**
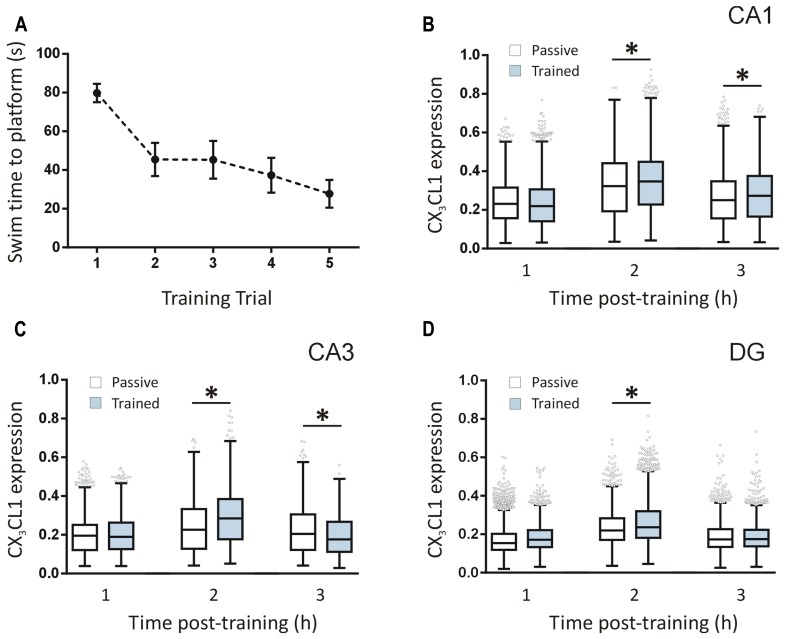
**Temporal changes in CX_3_CL1 expression in the rat hippocampus post-spatial learning measured by immunofluorescence. (A)** Graph shows the average latency-to-platform times for rats trained in a single session (five trials) of the water maze task (*n* = 12). **(B–D)** Box plots represent the distributions of fluorescence intensity values for CX_3_CL1 protein expression in CA1, CA3, and DG cells at 1, 2 and 3 h post-water maze training, compared to passive control animals. Changes in CX_3_CL1 expression were analyzed using Kruskal–Wallis ANOVA and Dunn’s multiple comparisons *post hoc* tests; ^∗^*p* < 0.01. There were four rats per time-point and three hippocampal sections analyzed per animal. The numbers of cells analyzed per hippocampal region were as follows: CA1: 1116–1201 cells; CA3: 1090–1247 cells; DG: 2888–3455 cells. The numbers of cells analyzed per time-point were: 1 h: 11,526 cells; 2 h: 10,355 cells; 3 h: 10,867 cells.

Rats were trained in a five-trial water maze session (*n* = 12 in total; *n* = 4 per time point) and the latencies to find the platform were recorded. Latency-to-platform times decreased significantly (one-way ANOVA, *p* < 0.001) over the five trials of the training session indicating that the animals acquired the task (**Figure 2A**). We quantified CX_3_CL1 expression in the hippocampus of these rats using immunofluorescence. Total CX_3_CL1 expression was very similar in trained and passive animals 1 h following training in all hippocampal regions (**Figures 2B–D**). At the 2 h time-point, CX_3_CL1 expression was significantly higher in trained animals compared to passive controls in all three hippocampal regions analyzed (Kruskal–Wallis ANOVA, Dunn’s multiple comparisons *post hoc* test, *p* < 0.001). At the 3 h time-point in the DG, CX_3_CL1 expression in trained animals again matched that of passive controls, highlighting the temporal specificity and transient nature of the 2 h up-regulation in dentate granule cells of rats that learned the spatial task. In the CA3, however, there was a significant, training-specific down-regulation in CX_3_CL1 expression whereas levels of CX_3_CL1 remained elevated in the CA1 region of trained animals compared to passive control counterparts at the 3 h time-point (Kruskal–Wallis ANOVA, Dunn’s multiple comparisons *post hoc* test, *p* < 0.01). It should be noted that passive control animals exhibited an up-regulation in CX_3_CL1 at the 2 h post-swim time-point indicating that, in addition to the learning-specific regulations described above, this chemokine is also responsive to the general stressors associated with the paradigm.

## CX_3_CL1 IS UP-REGULATED 2 h POST-THETA BURST STIMULATION IN DENTATE GRANULE NEURONS

**FIGURE 3:**
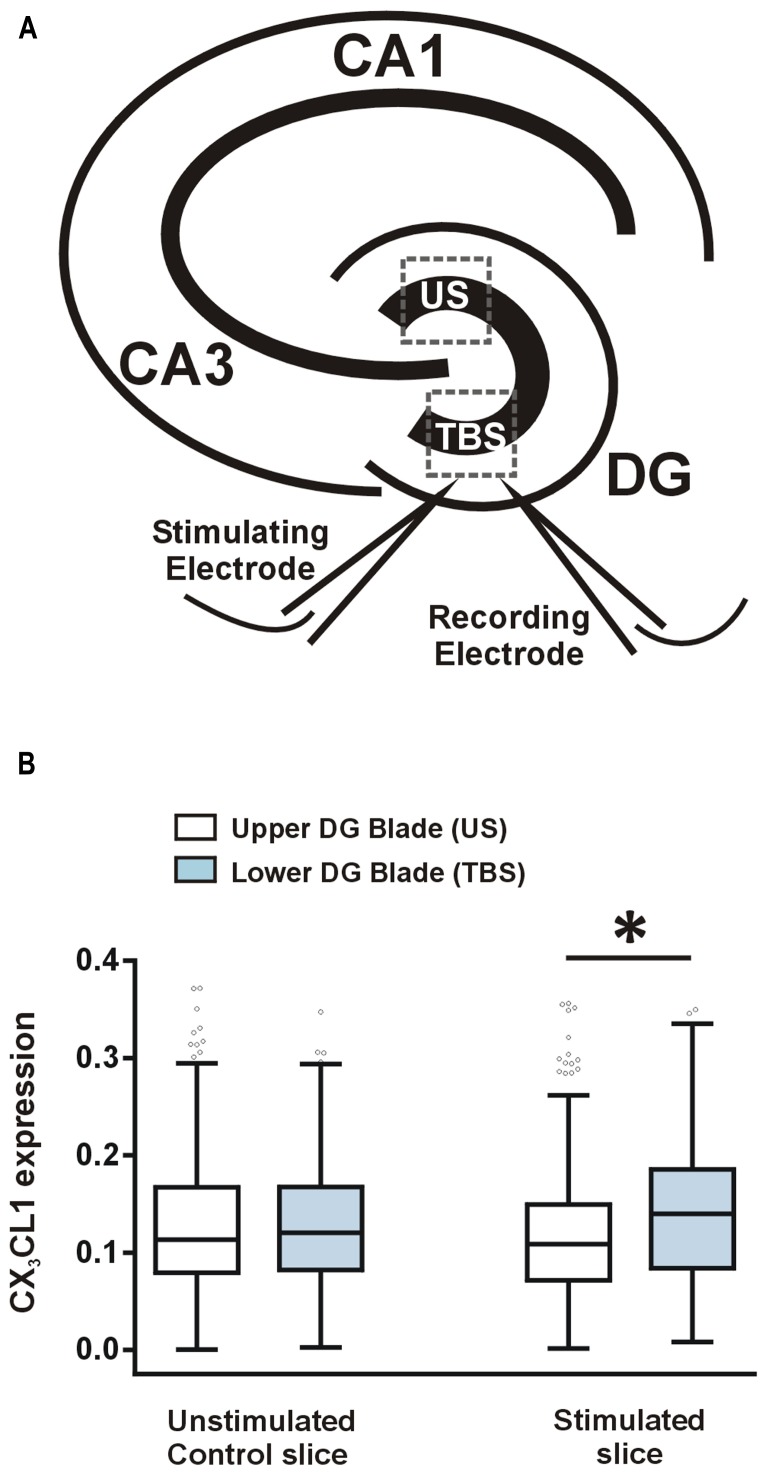
**CX_3_CL1 expression increases 2 h following theta-burst stimulation-induced LTP in the DG of acute hippocampal slices. (A)** Schematic diagram illustrating the position of stimulating and recording electrodes in hippocampal LTP experiments. Electrodes were always placed in the medial molecular layer of the lower blade of the DG approximately 100–200 μm apart. US, unstimulated DG blade; TBS, theta-burst stimulated blade. **(B)** Box plots showing the distribution of CX_3_CL1 fluorescence intensities in the upper and lower blades of the DG in stimulated slices and time-matched control slices. Asterisk indicates significant difference from the US blade (Kruskal–Wallis ANOVA and Dunn’s multiple comparisons *post hoc* tests; *p* < 0.001).

After measuring a learning-specific up-regulation in CX_3_CL1 in pyramidal and dentate granule neurons 2 h post-water maze-training, we next asked whether CX_3_CL1 is up-regulated after LTP-inducing TBS in acute hippocampal slices. TBS was delivered to the medial perforant path of the lower DG blade, as illustrated in **Figure 3A**. LTP was induced and recorded for 2 h, after which slices were snap-frozen. CX_3_CL1 expression on stimulated dentate granule cells was compared to that on neurons located in the upper unstimulated blade (US) of the DG (**Figure 3A**). LTP-inducing TBS resulted in an up-regulation in CX_3_CL1 expression after 2 h on the stimulated DG blade compared to the upper unstimulated DG blade (**Figure 3B**). No such difference between upper and lower blades was observed in time-matched control slices which received no stimulation.

### EFFECTS OF CX_3_CL1 ON LTP IN THE DENTATE GYRUS DEPENDS ON INHIBITORY TONE AND GABA_A_ RECEPTORS

**FIGURE 4:**
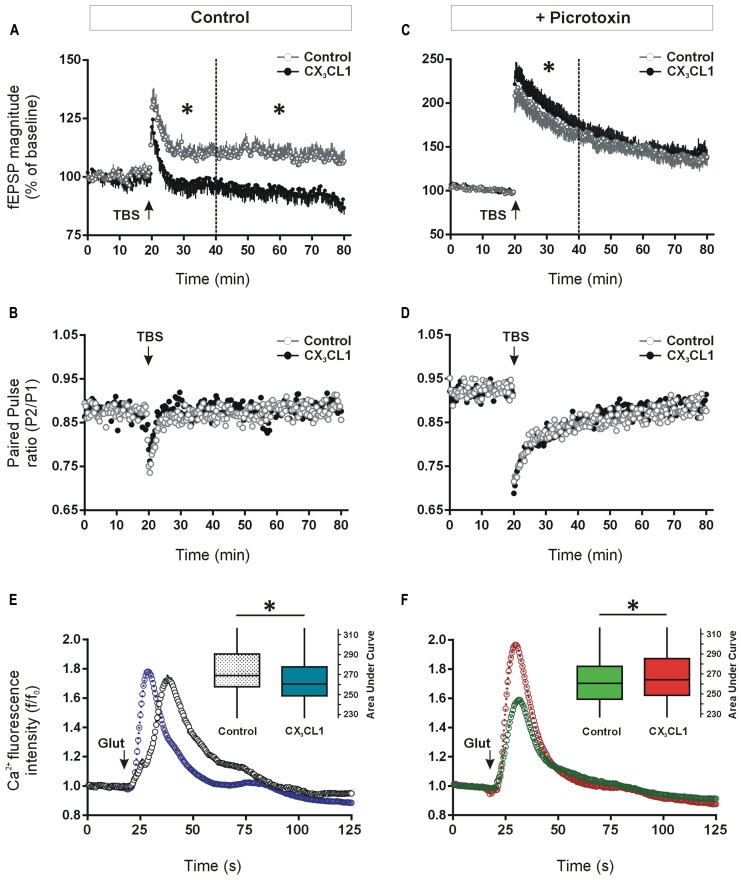
**Differential effects of CX_3_CL1 on long-term potentiation (LTP) in the DG and glutamate-induced intracellular calcium rise in the CA1, in the presence and absence of GABA_A_/chloride receptor blockade. (A)** The effect of the chemokine domain of CX_3_CL1 on LTP in acute hippocampal slices as measured by augmented field EPSP magnitude in the DG following theta-burst stimulation (TBS: 8 × 8 × 200 Hz). CX_3_CL1 inhibited dentate LTP in both the early and late phases post-TBS (i.e., 20–40 min and 40–80 min, respectively; one-way ANOVA; *p* < 0.05, indicated by an asterisk; *n* = 8 slices per group). CX_3_CL1 (500 pM) was present for the duration of the time period shown. **(B)** Shows the paired-pulse ratio between the first and second stimulations (50 ms interval) in the LTP experiment in **A**. **(C)** Shows the effect of CX_3_CL1 on dentate LTP in acute hippocampal slices as measured by augmented field EPSP magnitude following TBS in the presence of picrotoxin (100 μM). CX_3_CL1 (500 pM) and picrotoxin were present for the duration of the time period shown. CX_3_CL1 enhanced early LTP (one-way ANOVA; *p* < 0.05, indicated by an asterisk; *n* = 8 slices per group) while having no effect on late LTP. **(D)** Shows the paired-pulse ratio between the first and second stimulations (50 ms interval) in the LTP experiment in **C**. **(E)** Shows the effect of CX_3_CL1 on glutamate-induced calcium influx in the CA1 region of organotypic hippocampal slices cultured for 21 DIV. Pre-treatment of slice cultures with CX_3_CL1 (500 pM) for 15 min prior to glutamate exposure reduced calcium influx in the CA1 region (Mann–Whitney *U* test; *p* < 0.001). Box plot inset shows the area under the curve (AUC) for the whole experimental time-course. CX_3_CL1 (500 pM) was present for the duration of the time period shown. **(F)** The effect of CX_3_CL1 on glutamate-induced calcium influx in the CA1 region of organotypic hippocampal slices in the presence of picrotoxin. Pre-treatment of slice cultures with CX_3_CL1 (500 pM) and picrotoxin (100 μM) versus picrotoxin alone (control) for 15 min prior to glutamate exposure enhanced calcium influx in the CA1 region (Mann–Whitney *U* test; *p* < 0.001). Box plot inset shows the area under the curve (AUC) for the whole experimental time-course. CX_3_CL1 (500 pM) and/or picrotoxin (100 μM) were present for the duration of the time period shown.

We investigated a possible functional role for CX_3_CL1 up-regulation in modulating synaptic transmission during memory-associated synaptic plasticity. Previous studies have shown that CX_3_CL1 inhibits LTP and mimics long-term depression (LTD) at the CA3–CA1 synapses in acute hippocampal slices (Bertollini et al., 2006; Ragozzino et al., 2006; Maggi et al., 2009). Given that we observed memory-associated increases in CX_3_CL1 levels in CA1, CA3, and DG regions of the hippocampus following spatial learning, we next asked what effect CX_3_CL1 exerts on LTP in the dentate granule cell synaptic field. Pre-treatment with CX_3_CL1 (500 pM) significantly reduced induction and completely prevented maintenance of LTP in the DG (**Figure 4A**).

Consistent with previous work on pyramidal hippocampal neurons (Ragozzino et al., 2006), the paired-pulse ratio of DG neuron responses evoked by two successive stimuli (50 ms apart) was unaffected by CX_3_CL1 treatment either at baseline or following TBS (**Figure 4B**). While not ruling out a presynaptic contribution, these data support a post-synaptic action for CX_3_CL1-mediated inhibition of fEPSP amplitude in the DG following TBS.

Recent evidence has shown that CX_3_CL1 reduces the activity of serotonergic neurons in the Raphe nucleus through enhanced GABA_A_ receptor-mediated inhibition (Heinisch and Kirby, 2009). We next investigated if CX_3_CL1-mediated control of glutamatergic neuroplasticity in the hippocampal DG requires GABAergic inhibitory transmission. LTP was induced in the presence of GABA_A_ receptor/chloride channel blocker picrotoxin (100 μM) using the same TBS protocol as in **Figure 4A**. As expected, the degree of potentiation of fEPSP amplitude was substantially greater in the presence of GABA_A_ receptor blockade (117 ± 5% versus 200 ± 3% of baseline average in first 10 min post-TBS in the absence and presence of picrotoxin, respectively; **Figures 4A,C**; Arima-Yoshida et al., 2011). Interestingly, CX_3_CL1 did not prevent LTP in the presence of picrotoxin (**Figure 4C**). In fact, when picrotoxin was present, the magnitude of LTP was enhanced by CX_3_CL1 during the initial 20 min following the TBS (*p* < 0.05). CX_3_CL1 again had no effect on paired-pulse depression in the presence of picrotoxin (**Figure 4D**) suggesting a post-synaptic action for CX_3_CL1-mediated short-term enhancement of fEPSP amplitude following TBS.

### EFFECTS OF CX_3_CL1 ON GLUTAMATE-INDUCED CALCIUM RESPONSES IN THE HIPPOCAMPUS REQUIRES GABA_A_ RECEPTOR ACTIVATION

Long-term potentiation of excitatory synaptic transmission in the hippocampus is heavily dependent on post-synaptic intracellular calcium rise (Bliss and Collingridge, 1993). We next assessed if CX_3_CL1 exerts differential effects on glutamate-induced intracellular calcium rise in the presence and absence of GABA_A_ receptor blockade. Live-cell calcium imaging was performed in organotypic hippocampal slice cultures pre-treated with CX_3_CL1 (500 pM) and/or picrotoxin (100 μM) for 15 min. Slices were then exposed to glutamate (30 μM) and intracellular calcium was monitored for 1 min 45 s in the CA1 pyramidal cell layer of the hippocampus. Pre-treatment with CX_3_CL1 prior to glutamate application resulted in an attenuated intracellular calcium rise in cells in the CA1 region of organotypic slice cultures (**Figure 4E**). While the peak amplitude of the calcium response in CX_3_CL1-treated slices was not different than in controls (Figure S2A), the total calcium entry in the 60 s post-glutamate exposure was reduced, as calculated by the area under the curve (AUC) in **Figure 4E**. In contrast, in the presence of GABA_A_ receptor/chloride channel blockade, CX_3_CL1 enhanced glutamate-induced calcium influx as measured by AUC (**Figure 4F**) or peak intracellular calcium (Figure S2B). These findings are consistent with the opposing actions of CX_3_CL1 on LTP studies in the presence and absence of picrotoxin. They suggest that neuronal responses to CX_3_CL1 can vary depending on the balance between excitation and inhibition in the hippocampal network. Therefore, during periods of enhanced excitatory activity in the hippocampus, CX_3_CL1 may act as a neuroprotective dampener of excessive glutamatergic neurotransmission and this action appears to be dependent on GABA-mediated inhibition.

### CX_3_CL1 INHIBITS GLUTAMATE-INDUCED CALCIUM DYNAMICS IN BOTH NEURONS AND GLIAL CELL TYPES

**FIGURE 5:**
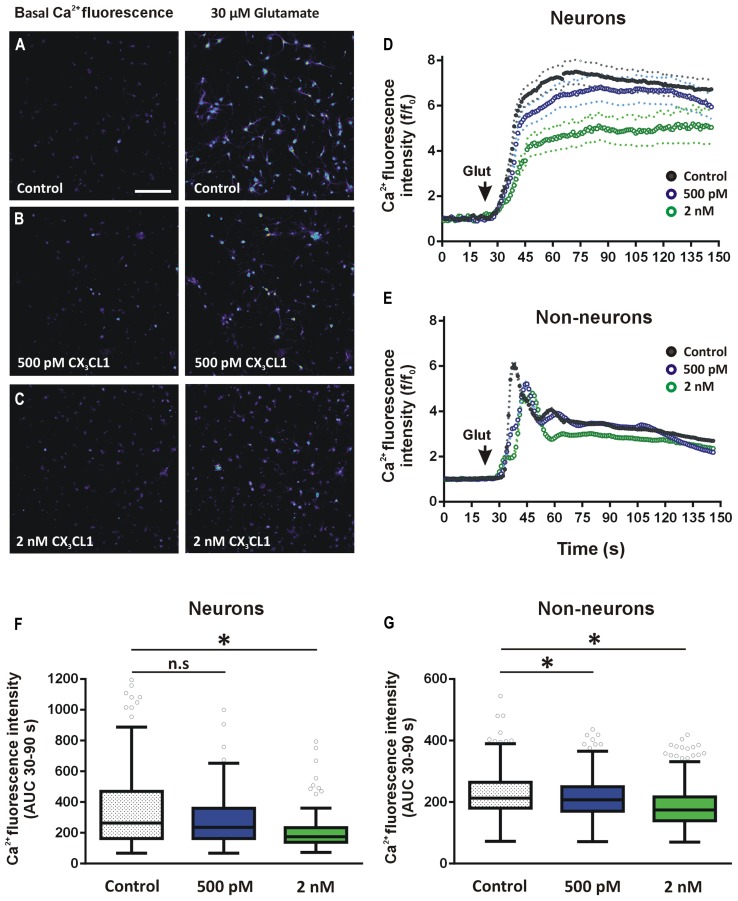
**Effect of CX_3_CL1 on glutamate-induced calcium dynamics in neurons and glia. (A–C)** Representative images of intracellular calcium [Ca^2+^]_i_ levels in mixed neuron-glial primary hippocampal cultures before and after 30 μM glutamate exposure. Control cells **(A)** were untreated prior to glutamate exposure. Treated cells were exposed to either **(B)** 500 pM or **(C)** 2 nM CX_3_CL1 for 15 min. Scale bar = 200 μm. **(D,E)** Shows the time-course of the [Ca^2+^]_i_ response to glutamate in neurons **(D)** and non-neuronal cells **(E)**. Cells were treated for 15 min with either 500 pM (blue circles) or 2 nM (green circles) CX_3_CL1 for 15 min prior to 30 μM glutamate exposure. Untreated control time-course is represented by black circles. Relative changes in [Ca^2+^]_i_ were calculated for each cell at each time point as *f*/*f*_0_, where *f* is the [Ca^2+^]_i_ fluorescence in each frame and *f*_0_ is the average baseline fluorescence per cell, calculated 20 s prior to glutamate addition. Primary hippocampal cell cultures were divided into neurons (138, 171, and 101 cells for control, 500 pM and 2 nM CX_3_CL1-treated cells, respectively) and non-neuronal (432, 726, and 604 cells for control, 500 pM and 2 nM CX_3_CL1-treated cells, respectively) cell populations based on their [Ca^2+^]_i_ response to 30 μM glutamate (Pickering et al., 2008). **(F,G)** Quantification of the calcium imaging time-courses in **D** and **E**. 500 pM CX_3_CL1 attenuates glutamate-induced [Ca^2+^]_i_ increases in non-neuronal cell types (Kruskal–Wallis ANOVA and Dunn’s multiple comparisons *post hoc* tests; ^∗^*p* < 0.05), but has no significant effect on [Ca^2+^]_i_ response in neurons. Pre-treatment of hippocampal cell cultures for 15 min with 2 nM CX_3_CL1, however, significantly attenuates glutamate-induced [Ca^2+^]_i_ influx in both non-neuronal cells and in neurons (Kruskal–Wallis ANOVA and Dunn’s multiple comparisons *post hoc* tests; ^∗^*p* < 0.001). The results represent combined data from five to six individual cover-slips per treatment group and from two separate cell culturing days.

In order to assess if physiological concentrations of CX_3_CL1 exert equivalent effects on glutamate-induced calcium responses in both neurons and non-neuronal cell populations, we used hippocampal mixed cell culture preparation. Primary hippocampal cells were pre-treated with CX_3_CL1 (500 pM or 2 nM) for 15 min prior to glutamate (30 μM) challenge. Importantly, we have shown previously that using this mixed hippocampal cell culture preparation, we can discriminate between neurons and non-neuronal cells based on the shapes of their respective glutamate-mediated calcium response curves (Pickering et al., 2008). This allowed us to simultaneously evaluate the effects of different concentrations of CX_3_CL1 on intracellular calcium dynamics in neurons and non-neuronal cells following glutamate exposure (**Figures 5A–C**). This method of distinguishing between neurons and non-neuron cell types was shown to be as accurate as traditional methods of immunocytochemistry staining for neuronal and glial cell markers (NeuN and GFAP, respectively; Pickering et al., 2008). Neurons were identified by their substantial and prolonged increase of intracellular calcium following glutamate administration while non-neurons exhibited a sharp rise and fall back to plateaux (**Figures 5D,E**, respectively). In non-neuronal cells, both low (500 pM) and higher (2 nM) physiologically relevant concentrations of CX_3_CL1 attenuated glutamate-induced calcium influx, in a dose-dependent manner (Kruskal–Wallis ANOVA, Dunn’s multiple comparisons *post hoc* test, *p* < 0.05; **Figures 5E,G**). In neurons, however, the lower concentration of CX_3_CL1 (500 pM) had little or no effect on glutamate-induced calcium influx (**Figures 5D,F**; and Figure S2C). Pre-treatment of hippocampal cultures with a higher concentration (2 nM) of CX_3_CL1 caused a substantial attenuation of glutamate-mediated calcium responses in neurons (Kruskal–Wallis ANOVA, Dunn’s multiple comparisons *post hoc* test, *p* < 0.001; **Figures 5D,F**), in addition to causing further dose-dependent attenuations in calcium influx in non-neuronal cell types (**Figures 5E,G**; and Figure S2D). The differential responses of CX_3_CL1 on neurons and non-neurons at the lower concentration (500 pM) may relate to variations in absolute levels of CX_3_CR1 receptor expression on distinct cell populations in the hippocampus.

## DISCUSSION

CX_3_CL1 is among an increasing number of cytokines and chemokines implicated in both normal functions and pathophysiological conditions of the brain (White and Greaves, 2012; Mattison et al., 2013; Sheridan and Murphy, 2013; Wu et al., 2013; Briones et al., 2014). In the present study, we identified a transient up-regulation of CX_3_CL1 production in the hippocampus 2 h following spatial learning or induction of LTP. Importantly, these up-regulations were specific to either the memory encoding process or the TBS since the up-regulations measured were compared directly to swim-matched passive controls or unstimulated dentate granule cells, respectively. In addition, we did observe a 2 h up-regulation in CX_3_CL1 in the passive control group of animals, indicating that this chemokine may also be responsive to stress and anxiogenic environmental conditions. However, the significant training-specific up-regulations in CX_3_CL1, over and above those measured in passive control animals at the 2 h time-point, implicate CX_3_CL1 in memory-related synaptic plasticity in the hippocampus. The enhanced glutamate neurotransmission and resultant increase in hippocampal neuronal activity associated with both spatial memory formation and LTP are the most likely drivers of the production of CX_3_CL1 in our studies. CX_3_CL1 has been reportedly up-regulated in several settings of augmented glutamatergic transmission where the chemokine is clearly protective against excitotoxic cell death (Tong et al., 2000; Tarozzo et al., 2002; Limatola et al., 2005; Cipriani et al., 2011; Briones et al., 2014). Moreover, both CX_3_CL1 and its receptor, CX_3_CR1, have been shown to be up-regulated in hippocampal neurons after pilocarpine-induced status epilepticus (Yeo et al., 2011), a condition characterized by excessive glutamatergic excitation. Interestingly, 3 h post-learning, we observed opposing regulations in CX_3_CL1 in the CA1 and CA3 pyramidal cell layers. This may relate to distinct plasticity mechanisms and/or distinct functions of these hippocampal subregions during spatial memory tasks (Hunsaker and Kesner, 2008; Rolls, 2010). Overall, the abundance and expression pattern of CX_3_CL1 make the chemokine ideally suited for sensing hippocampal glutamate tone. Our data supports a possible role for CX_3_CL1 in homeostatic mechanisms of synaptic scaling during memory-associated synaptic plasticity.

Previous studies have reported that CX_3_CL1 inhibits LTP and induces LTD-like effects in the CA1 region of the hippocampus (Bertollini et al., 2006; Ragozzino et al., 2006; Maggi et al., 2009). We found a matching inhibitory action of CX_3_CL1 on LTP in the DG. These observations are in good agreement with CX_3_CL1-mediated inhibition of glutamatergic synaptic activity of hippocampal neurons (Ragozzino et al., 2006). CX_3_CL1-mediated regulation of glutamate transmission has been shown to be due to its post-synaptic effects on neurons and involves the dephosphorylation of the GluR1 AMPA receptor subunit on serine 845, a mechanism reminiscent of LTD (Ragozzino et al., 2006). LTD can play vital roles in the context of memory-associated synaptic plasticity including synaptic scaling and enhanced signal-to-noise ratio mechanisms.

The GABA_A_ receptor/chloride channel blocker, picrotoxin, used in the current studies would decrease GABAergic inhibitory transmission promoting depolarization in some cells with corresponding increases in intracellular calcium (Antonucci et al., 2012). Thus, the current work suggests GABA_A_ receptor/chloride channel activity within the hippocampal neuronal network must be intact for CX_3_CL1 to attenuate glutamatergic neurotransmission or LTP. This situation is remarkably similar to that described for CX_3_CL1 inhibition of serotonergic neurons of the Raphe nucleus (Heinisch and Kirby, 2009). While this effect may relate to basal inhibitory tone, CX_3_CL1 could actually be enhancing GABA_A_ receptor function. For example, CX_3_CL1 enhances phosphorylation and activation of Akt in neurons and this serine/threonine kinase has been associated with phosphorylation of the GABA_A_ beta2 receptor subunit, a modification that enhances activity of the receptor (Meucci et al., 2000; Wang et al., 2003). Moreover, several signaling systems can enhance GABA-mediated inhibition through promotion of rapid insertion of GABA_A_ receptors into the post-synaptic plasma membrane (Wan et al., 1997; Nusser et al., 1998; Mizoguchi et al., 2003; Jovanovic et al., 2004).

When discussing potential mechanisms by which CX_3_CL1 influences neurons we must to mindful of the inconsistencies in the literature with regard to the expression of the CX_3_CR1 receptor by neurons (Meucci et al., 1998, 2000; Maciejewski-Lenoir et al., 1999; Jung et al., 2000; Hatori et al., 2002; Hughes et al., 2002; Tarozzo et al., 2003; Deiva et al., 2004; Verge et al., 2004; Limatola et al., 2005; Cardona et al., 2006; Zhuang et al., 2007; Heinisch and Kirby, 2009; Ruchaya et al., 2012, 2014). The consensus from work with the CX_3_CR1^-/-^-GFP knock-in mouse (Cardona et al., 2006) suggests that CX_3_CR1 expression is restricted to microglial cells in the CNS under naïve conditions *in vivo*. Studies of CX_3_CR1 expression in rats, however, have found evidence of receptor expression on neuronal cell types in various brain regions (Heinisch and Kirby, 2009; Ruchaya et al., 2012, 2014). Here, we report rapid modulatory effects of CX_3_CL1 on neuronal events in hippocampal tissue. Our data does not conclusively support a direct action of CX_3_CL1 on neurons and we cannot discount the possibility that the effects we see on LTP and calcium influx in neuronal cell types happen as a consequence of CX_3_CL1-mediated activation of CX_3_CR1 solely on microglial cell types. If this is true, however, the ability of microglia to rapidly regulate multiple hippocampal memory-associated synaptic plasticity processes may be much more extensive than traditionally thought. The evidence from rats that CX_3_CR1 is expressed on hippocampal neuron allows the possibility that, as is the case for CX_3_CL1-mediated regulation of serotonergic neurons of the dorsal Raphe, glutamatergic neuron-derived CX_3_CL1 may act in an autocrine/paracrine fashion in the hippocampus during periods of synaptic plasticity to regulate glutamate-mediated neurotransmission tone.

While previous studies have shown that CX_3_CL1 causes an increase in intracellular calcium in neurons and other cell types (Oh et al., 2002; Deiva et al., 2004; Ragozzino et al., 2006) this effect of CX_3_CL1 becomes apparent at concentrations of 25 nM and higher (Oh et al., 2002; Deiva et al., 2004), well above the levels we investigated here and we observed no such increase in intracellular calcium. At a concentration of 2 nM, in both neurons and non-neurons, CX_3_CL1 pre-incubation suppressed glutamate-mediated rises in intracellular calcium levels. These data are in good agreement with previous findings of a protective effect of inflammatory levels of CX_3_CL1 against glutamate excitotoxicity and glutamate NMDA receptor activation, in particular (Meucci et al., 1998; Deiva et al., 2004; Limatola et al., 2005), although, unlike the current work, the latter effect was linked to increased intracellular calcium (Ragozzino et al., 2006). Calcium oscillations in both neuronal and non-neuronal cells are important for cellular growth, migration and synaptic structural refinement (Katz and Shatz, 1996; Komuro and Rakic, 1998; Spitzer et al., 2000). Thus, the dampening of such signaling by CX_3_CL1 is suggestive of a role in stability of network connectivity and activity. Overall, the current data along with information in the published literature reveal that a role for CX_3_CL1 in control of glutamate-mediated excitatory neurotransmission during excitotoxic events can be extended to situations of synaptic plasticity required for normal functions such as memory formation.

An increasing number of chemokines exhibit extensive regulation across a range of situations where neuronal plasticity is involved; including memory-associated functional plasticity, protective plasticity in the setting of ischemia and maladaptive plasticity such as that underpinning neuropathic pain (Adler et al., 2006; Rostene et al., 2007; McAfoose and Baune, 2009; Old and Malcangio, 2012). Specifically, the role for CX_3_CL1 in control of hippocampus and Raphe activity suggests that infection-, inflammation-, and/or chronic disease-associated increases in the chemokine could contribute to reduced hippocampal and Raphe output, alterations that could, in turn, precipitate depressed mood and heightened anxiety among other disorders of brain function (Meltzer, 1990; Bast, 2011; Small et al., 2011). The extensive expression of CX_3_CL1 on neurons in the adult hippocampus and CX_3_CL1 up-regulation post-spatial learning supports a direct role for CX_3_CL1 in memory-associated synaptic plasticity. To better understand the role of CX_3_CL1 up-regulation following learning it will be important to assess the consequences of blocking such chemokine signaling on learning and memory function.

## Conflict of Interest Statement

The authors declare that the research was conducted in the absence of any commercial or financial relationships that could be construed as a potential conflict of interest.

## SUPPLEMENTARY MATERIAL

**Figure S1 | Image analysis automation using EBImage software. (A)** 40× magnification of the apex of the hippocampal dentate gyrus. Green: CX_3_CL1; Red: NeuN and Blue: Hoechst. Scale bar = 50 μm. **(B)** Hoechst channel alone. Stains cell nuclei. **(C)** NeuN channel alone. Labels neuronal cell bodies. **(D)** CX_3_CL1 channel alone. Labels CX_3_CL1 protein on dentate granule cells. **(E)** The blue fluorescence intensity was thresholded in order to select only Hoechst-stained nuclei (white). **(F)** Using a morphological kernel expansion algorithm, nuclei were dilated to select a cell soma region surrounding each nucleus. **(G)** A distance map was generated which calculates the distance each foreground (white) pixel is from the nearest background (black) pixel. **(H)** The watershed segmentation algorithm was then employed in order to separate all cell bodies from one another. The varying shades of gray in the image denote separated cells.

**Figure S2 | Effect of CX_**3**_CL1 on the peak glutamate-induced calcium response in organotypic slice cultures and mixed hippocampal cell cultures. (A)** Shows the effect of CX_3_CL1 on glutamate-induced calcium influx in the CA1 region of organotypic hippocampal slices cultured for 21 DIV. Pre-treatment of slice cultures with CX_3_CL1 (500 pM) for 15 min prior to glutamate (30 μM) exposure had no effect on the peak Ca^2+^ fluorescence intensity achieved by each cell (Mann–Whitney *U* test; *p* < 0.001). **(B)** Shows the effect of CX_3_CL1 on glutamate-induced calcium influx in the CA1 region of organotypic hippocampal slices in the presence of picrotoxin. Pre-treatment of slice cultures with CX_3_CL1 (500 pM) and picrotoxin (100 μM) versus picrotoxin alone (control) for 15 min prior to glutamate (30 μM) exposure enhanced the peak Ca^2+^ fluorescence intensity achieved by each cell (Mann–Whitney *U* test; *p* < 0.001). **(C)** Shows the effect of CX_3_CL1 (500 pM and 2 nM) on glutamate-induced calcium influx in neuronal cell types within mixed hippocampal cell cultures. Pre-treatment of primary hippocampal cell cultures with the lower dose of CX_3_CL1 (500 pM) for 15 min prior to glutamate (30 μM) exposure had no effect on the peak Ca^2+^ fluorescence intensity achieved by each neuronal cell (Kruskal–Wallis ANOVA and Dunn’s multiple comparisons *post hoc* tests; *p* < 0.001). The higher dose of CX_3_CL1 (2 nM), however, attenuated the peak Ca^2+^ fluorescence intensity achieved by neurons in response to glutamate application. **(D)** Shows the effect of CX_3_CL1 (500 pM and 2 nM) on glutamate-induced calcium influx in non-neuronal cell types within mixed hippocampal cell cultures. Pre-treatment of primary hippocampal cell cultures with the lower dose of CX_3_CL1 (500 pM) for 15 min prior to glutamate (30 μM) exposure had no effect on the peak Ca^2+^ fluorescence intensity achieved by each non-neuronal cell (Kruskal–Wallis ANOVA and Dunn’s multiple comparisons *post hoc* tests; *p* < 0.001). The higher dose of CX_3_CL1 (2 nM), however, attenuated the peak Ca^2+^ fluorescence intensity achieved by each non-neuronal cell in response to glutamate application.
