# Supplementary material for: CX3CL1 is up-regulated in the rat hippocampus during memory-associated synaptic plasticity
Source: Front Cell Neurosci. 2014 Aug 12;8:233. doi: 10.3389/fncel.2014.00233 (PMC4130185; doi:10.3389/fncel.2014.00233)
Supplement: Supplementary file 1 [file Presentation_1.PDF]

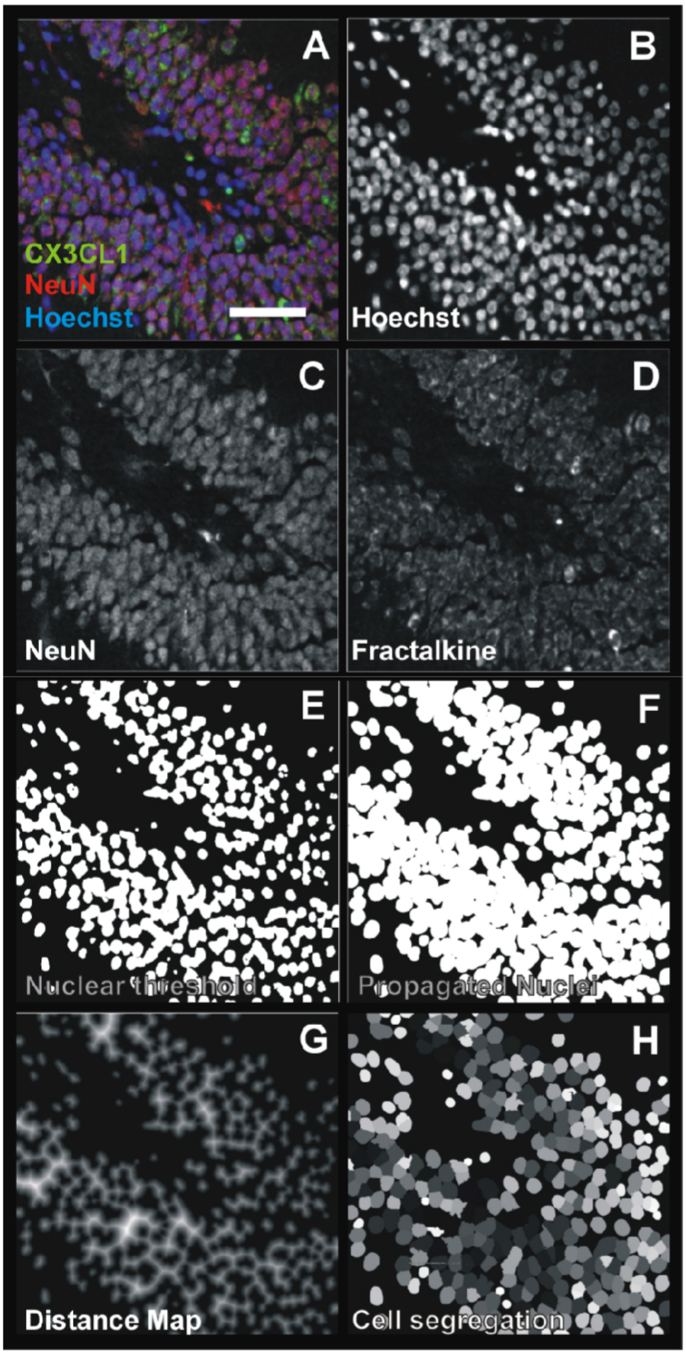

Supplemental Figure 1: Image analysis automation using EBImage software. (A) 40X magnification of the apex of the hippocampal dentate gyrus. Green: CX3CL1; Red: NeuN and Blue: Hoechst. Scale bar = 50 µm. (B) Hoechst channel alone. Stains cell nuclei. (C) NeuN channel alone. Labels neuronal cell bodies. (D) CX3CL1 channel alone. Labels CX3CL1 protein on dentate granule cells. (E) The blue fluorescence intensity was thresholded in order to select only Hoechst-stained nuclei (white). (F) Using a morphological kernel expansion algorithm, nuclei were dilated to select a cell soma region surrounding each nucleus. (G) A distance map was generated which calculates the distance each foreground (white) pixel is from the nearest background (black) pixel. (H) The watershed segmentation algorithm was then employed in order to separate all cell bodies from one another. The varying shades of grey in the image denote separated cells.
