# Supplementary material for: CX3CL1 is up-regulated in the rat hippocampus during memory-associated synaptic plasticity
Source: Front Cell Neurosci. 2014 Aug 12;8:233. doi: 10.3389/fncel.2014.00233 (PMC4130185; doi:10.3389/fncel.2014.00233)
Supplement: Supplementary file 2 [file Presentation_2.PDF]

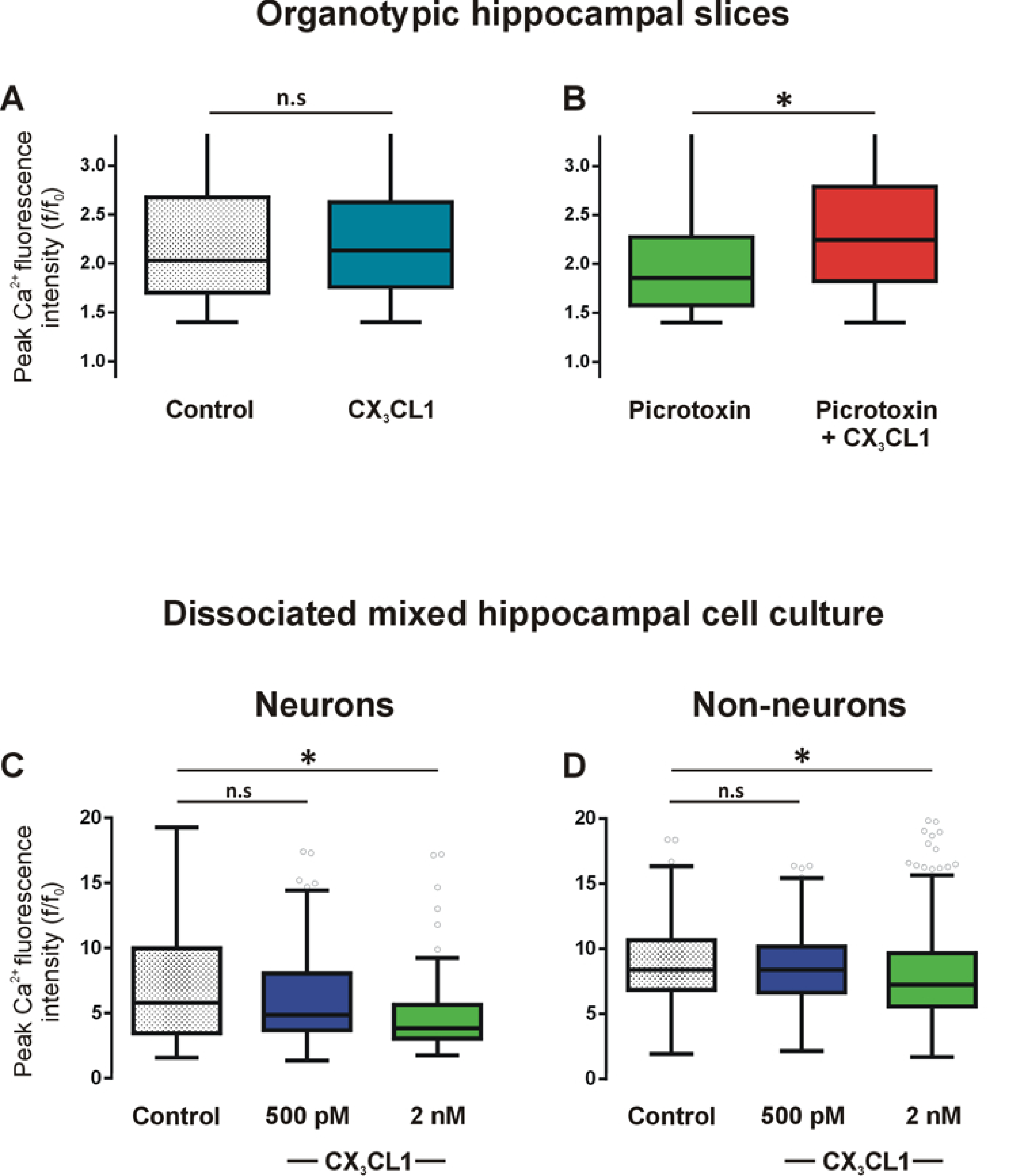
Organotypic hippocampal slices
A
n.s
Peak Ca2+ fluorescence intensity (f/f0)
3.0
2.5
2.0
1.5
1.0
Control
CX3CL1
B
*
Picrotoxin
Picrotoxin + CX3CL1
Dissociated mixed hippocampal cell culture
Neurons
Non-neurons
C
*
n.s
20
15
10
5
0
Control
500 pM
2 nM
— CX3CL1 —
D

Supplemental Figure 2: Effect of CX3CL1 on the peak glutamate-induced calcium response in organotypic slice cultures and mixed hippocampal cell cultures. (A) Shows the effect of CX3CL1 on glutamate-induced calcium influx in the CA1 region of organotypic hippocampal slices cultured for 21 DIV. Pre-treatment of slice cultures with CX3CL1 (500 pM) for 15 min prior to glutamate (30 μM) exposure had no effect on the peak Ca2+ fluorescence intensity achieved by each cell (Mann–Whitney U test; $p < 0.001$). (B) Shows the effect of CX3CL1 on glutamate-induced calcium influx in the CA1 region of organotypic hippocampal slices in the presence of picrotoxin. Pre-treatment of slice cultures with CX3CL1 (500 pM) and picrotoxin (100 μM) versus picrotoxin alone (control) for 15 min prior to glutamate (30 μM) exposure enhanced the peak Ca2+ fluorescence intensity achieved by each cell (Mann–Whitney U test; $p < 0.001$). (C) Shows the effect of CX3CL1 (500 pM and 2 nM) on glutamate-induced calcium influx in neuronal cell types within mixed hippocampal cell cultures. Pre-treatment of primary hippocampal cell cultures with the lower dose of CX3CL1 (500 pM) for 15 min prior to glutamate (30 μM) exposure had no effect on the peak Ca2+ fluorescence intensity achieved by each neuronal cell (Kruskal-Wallis ANOVA and Dunn's multiple comparisons post-hoc tests; $p < 0.001$). The higher dose of CX3CL1 (2 nM), however, attenuated the peak Ca2+ fluorescence intensity achieved by neurons in response to glutamate application. (D) Shows the effect of CX3CL1 (500 pM and 2 nM) on glutamate-induced calcium influx in non-neuronal cell types within mixed hippocampal cell cultures. Pre-treatment of primary hippocampal cell cultures with the lower dose of CX3CL1 (500 pM) for 15 min prior to glutamate (30 μM) exposure had no effect on the peak Ca2+ fluorescence intensity achieved by each non-neuronal cell (Kruskal-Wallis ANOVA and Dunn's multiple comparisons post-hoc tests; $p < 0.001$). The higher dose of CX3CL1 (2 nM), however, attenuated the peak Ca2+ fluorescence intensity achieved by each non-neuronal cell in response to glutamate application.
